# Supplementary material for: Conditional chemoconnectomics (cCCTomics) as a strategy for efficient and conditional targeting of chemical transmission
Source: eLife. 2024 Apr 30;12:RP91927. doi: 10.7554/eLife.91927 (PMC11060718; doi:10.7554/eLife.91927)
Supplement: Figure 3—source data 3. [file elife-91927-fig3-data3.zip › Figure 3 source data3 part 2.zip!/Figure 3-Source data 3-part2/1053-1-CRISPResso_Report_mpeqg9da.zip!/CRISPResso_on_1053_4/4c.Quantification_window_insertion_deletion_substitution_locations.pdf]

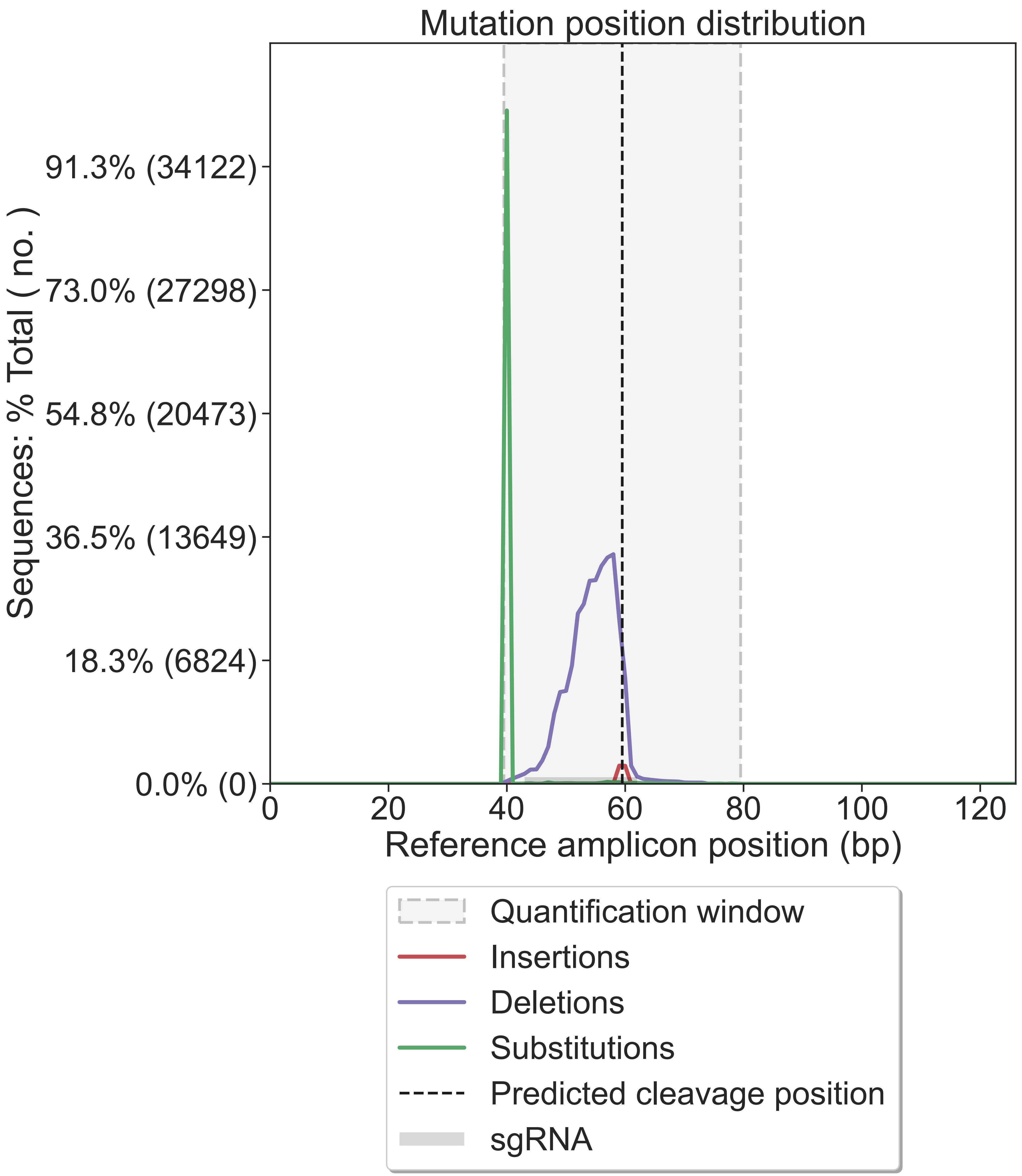
Mutation position distribution
91.3% (34122)
73.0% (27298)
54.8% (20473)
36.5% (13649)
18.3% (6824)
0.0% (0)
Sequences: % Total ( no. )
0
20
40
60
80
100
120
Reference amplicon position (bp)
Quantification window
Insertions
Deletions
Substitutions
Predicted cleavage position
sgRNA
